# Phylogeny, Seed Trait, and Ecological Correlates of Seed Germination at the Community Level in a Degraded Sandy Grassland

**DOI:** 10.3389/fpls.2016.01532

**Published:** 2016-10-17

**Authors:** Zhengning Wang, Lixin Wang, Zhimin Liu, Yanjuan Li, Qingqing Liu, Bo Liu

**Affiliations:** ^1^College of Forestry, Fujian Agriculture and Forestry UniversityFuzhou, China; ^2^Engineering Research Center of Chinese Fir, State Forestry AdministrationFuzhou, China; ^3^Department of Earth Sciences, Indiana University-Purdue University, IndianapolisIndianapolis, IN, USA; ^4^State Key Laboratory of Forest and Soil Ecology, Institute of Applied Ecology, Chinese Academy of SciencesShenyang, China

**Keywords:** seed dispersal, seed mass, seed shape, life form, phylogeny

## Abstract

Seed germination strongly affects plant population growth and persistence, and it can be dramatically influenced by phylogeny, seed traits, and ecological factors. In this study, we examined the relationships among seed mass, seed shape, and germination percentage (GP), and assessed the extent to which phylogeny, seed traits (seed mass, shape, and color) and ecological factors (ecotype, life form, adult longevity, dispersal type, and onset of flowering) influence GP at the community level. All analyses were conducted on the log-transformed values of seed mass and arcsine square root-transformed values of GP. We found that seed mass and GP were significantly negatively correlated, whereas seed shape and GP were significantly positively correlated. The three major factors contributing to differences in GP were phylogeny, dispersal type, and seed shape (explained 5.8, 4.9, and 3.1% of the interspecific variations independently, respectively), but GP also influenced by seed mass and onset of flowering. Thus, GP was constrained not only by phylogeny but also by seed traits and ecological factors. These results indicated that GP is shaped by short-term selective pressures, and long-term phylogenetic constrains. We suggest that correlates of phylogeny, seed traits, and ecology should be taken into account in comparative studies on seed germination strategies.

## Introduction

Seed germination, a central seed trait, is a key bottleneck in plant life history, potentially affecting the distribution and abundance of species in communities (Guo et al., 2000; Souza and Fagundes, 2014). Most life history traits are correlated (Moles and Leishman, 2008; Norden et al., 2009), and some traits may affect seed germination percentage. Understanding how life-history traits are related to germination percentage is crucial for understanding the evolution and ecology of seed germination strategies. Although relationships between life-history traits and germination have received considerable attention, most studies have focused on individual species without scaling up to a community level (but see Liu et al., 2007; Xu et al., 2014). Furthermore, variation in germination strategies among species in a community may support higher diversity by allowing temporal partitioning of environmental resources and providing a buffer against extinction (Chesson, 2000; Gremer and Venable, 2014). Therefore, understanding germination strategies of seeds within a single community has implications for trait evolution as well as population and community dynamics.

Seed mass has long been regarded as an important aspect of plant reproductive biology (Baker, 1972; Moles et al., 2005, 2007). Seed mass influences many different life-history stages in plants. For instance, small-seeded species have low rates of seedling survivorship (Moles and Westoby, 2004), and they tend to have high seed output but short lifespans (Moles and Westoby, 2004). Relationships between seed mass and germination are less well understood than relationships between seed mass and other life-history stages. Differences in seed mass among species are due in part to different levels of starch and endosperm nutrients, and may influence germination percentage (Chen et al., 2002; Soriano et al., 2011). Studies have shown that seed mass may affect germination percentage (Reich, 1994; Paz et al., 1999; Bu et al., 2007; Xu et al., 2014). Regardless, it is widely accepted that large seeds generally have a higher germination percentage than small seeds. However, there is little information on the relationship between seed mass and germination in arid or semi-arid ecosystems, especially, the degraded grasslands in arid environments.

Seed shape, which varies in three dimensions, is another important trait that affects germination (Weiher et al., 1999; Tekrony et al., 2005). Theoretical studies have predicted that species with elongate and flat seeds should germinate more frequently than those with round seeds (Grime et al., 1981). Elongate and flat seeds are more likely to experience post-dispersal predation than round seeds, and should therefore show prompt germination to avoid risks of mortality. However, strong empirical data supporting this prediction are lacking (Liu et al., 2007).

It is not yet fully known how seed germination percentage is related to various factors. Germinability has been predicted to be associated with phylogeny, seed traits, and ecological factors (Bu et al., 2007; Norden et al., 2009). These hypotheses can be tested by answering two questions. Which factors of phylogeny, seed traits, and ecology affect seed germination? Which factors are most significant in explaining seed germination percentage? Although there has been research on the relationship between seed traits and germinability, there is limited information on the proportion of germination variation among species that could be attributed to the species' phylogenetic background, seed traits, and ecological factors. The relative importance of phylogeny, seed traits such as mass, shape and color, and ecological factors such as ecotype, life form, adult longevity, dispersal type, and onset of flowering in determining seed germination percentage need to be determined for degraded sandy grassland ecosystems.

The Horqin Sandy Land located in the arid zone of Inner Mongolia, China, is a well-known grassland in northeastern China (Zhu and Chen, 1994; Wang et al., 2003). In the last few decades, it has been affected by desertification, likely the consequence of over-cultivation, intensive fire wood collection, and overgrazing. Mobile dunes, semi-stabilized dunes, and stabilized dunes are distributed over some areas of the steppe. In degraded sandy grasslands, seedlings are vulnerable to desiccation, long periods of drought, high temperatures, and overgrazing (Liu et al., 2007; Zhao et al., 2011). Seed germination strategies are, in theory, some of the most effective strategies for plants inhabiting arid environments, which are typically characterized by highly variable and unpredictable environment (Philippi, 1993). Plants inhabiting arid degraded sandy grasslands have developed different strategies for seed germination to respond to the novel environmental pressures via long-term natural selection (Li et al., 2006). However, in recent years, many studies have focused on the relationship between seed morphology and vegetation processes (Liu et al., 2007; Yan et al., 2009; Ma et al., 2010; Zhao et al., 2011), but the link between the germination and seed traits, ecological factors at community level in this highly disturbed and stressed environment has not been elucidated. Studies of the relationships between seed germination and phylogeny, seed traits, and ecological factors would provide insights into seed germination strategies in unpredictable environments, especially in degraded sandy grasslands.

In the present study, we assessed relationships between germination percentage and phylogeny, seed traits, and ecological factors for 109 species in a degraded sandy grassland at the community level. The following questions were addressed: (1) whether species with small seeds would have a higher germination percentage than species with larger seeds, (2) whether species with elongate and flat seeds would have a higher germination percentage than those with round seeds, and (3) what proportion of germination variation among species could be attributed to the species' phylogeny, seed traits (mass, shape, and color), and ecological factors (ecotype, life form, adult longevity, dispersal type, and onset of flowering).

## Materials and methods

### Study site

The study grassland is located in the Horqin Sandy Land in northeastern China (118° 35′–123° 30′ E, 42° 41′–45° 45′ N), which has a continental semiarid monsoonal climate with a very cold winter and a warm summer. Mean annual temperature is 6.3°C; the lowest and the highest monthly mean temperatures are −14.0°C in January and 23.0°C in July, respectively. Mean annual precipitation is 288 mm, of which 70–80% is received in June, July, and August. The prevailing winds are from the northwest from March to May and from the southeast from June to September. Annual mean wind velocity ranges from 3.0 to 4.4 m s^−1^. The soils are sandy, loose, and very susceptible to wind erosion. Ninety percent of the total area has been desertified. The landscape is characterized by mobile, semi-stabilized, and stabilized dunes.

### Seed collection

Mature seeds of 109 species growing in different habitats were collected from August to November (at the time of seed dispersal). Seeds were collected from 15 to 25 plants per species. Seeds from one species were thoroughly mixed to minimize the contribution from single plants. Air-dried seeds were stored in paper bags in the laboratory.

### Germination experiment

For each species, five replicates of 50 seeds were placed on filter paper in Petri dishes (9 cm diameter). The filter paper was moistened with distilled water and the dishes were placed in temperature and light-controlled incubators (14:10 h, light: dark cycle; photosynthetic photon flux density (PPFD) of 25–30 μmol m^−2^ s^−1^ at seed level). The daytime temperature was kept at 28°C and the night temperature was 16°C, to approximate the mean daily maximum and minimum temperature in 3–5-cm-deep soil during rainy days from May to August. Each dish was inspected daily, and germinated seeds were counted and removed. The seeds whose radicles had emerged were considered to have germinated. Counting continued until no germination occurred for five successive days. Seeds that had not germinated were checked for viability. Germination percentage (GP) was calculated as the percentage of viable seeds that germinated.

### Seed mass

Seed mass, a measure of size, was represented by the average weight of single seed (Liu et al., 2007). For each plant species, seed mass was the average air-dried weight of 100 seeds, calculated from five replicates.

### Seed shape

The seed shape was calculated as the variance of three main perpendicular dimensions, i.e., length, width, and height (Thompson et al., 1993; Moles et al., 2000). Seed dimension variance is a standard index to describe seed shape (Thompson et al., 1993). The variance was calculated as follows, using the average of 10 seeds for each species:

$$\text{variance =}\sqrt{\;\frac{{\left(1-\frac{\text{width}}{\text{length}}\right)}^{2}+{\left(1-\frac{\text{height}}{\text{length}}\right)}^{2}+{\left(1-\frac{\text{height}}{\text{width}}\right)}^{2}}{3}}$$

The above equation gives a value for seed shape, such that spherical seeds have a variance of 0 and elongated or flattened seeds have a variance up to 0.33 (Thompson et al., 1993). In other words, larger variance values are associated with flatter seeds and smaller values with rounder seeds.

### Statistical analysis

To determine to what extent phylogenetic, seed traits (mass, shape, and color), and ecological factors (ecotype, life form, adult longevity, dispersal type, and onset of flowering) influence seed germination, a one-way, two-way, and factorial analysis of variance (ANOVA) were conducted (Mazer, 1989, 1990; Figueroa and Armesto, 2001). The one-way ANOVAs estimated the effect of each factor on the GP variance independently. Two-way ANOVAs were performed on the same data to assess the interactions between each pair of factors. Factorial ANOVAs provided two types of information (Figueroa and Armesto, 2001): individual factor effects and association effects. We calculated the proportion of the variance contributed by each factor to the complete ANOVA model to assess the combined effects of all factors by performing a series of factorial ANOVAs that estimated the effects all factors but one (incomplete model). We compared each incomplete ANOVA with the complete factorial ANOVA. The difference between the proportion of the total sum of squares (ss) of the complete model (its *R*^2^) and the *R*^2^ of the incomplete model represented the proportion of the total ss explained by the removed factor. If, in the complete ANOVA, a given class variable has a lower *R*^2^ value than in the incomplete ANOVAs from which a different variable has been deleted, the increase in the *R*^2^ value of the first variable would be due to an association (or correlation) or strong interaction with the second variable (see Mazer, 1989, 1990). In other words, when two variables were strongly associated, the ss of each variable would account for a higher proportion of the total ss of the model when the other was not included in the model.

GP was arcsine square root transformed and seed masses were log transformed to improve normality and stabilize variances. All analyses were performed with SPSS 18.0 software.

We grouped species into the following categories prior to analysis:

**Phylogenetic group**. To examine effects of phylogeny on germination, the 109 angiosperm species were grouped by order according to the APG III system of plant classification (Angiosperm Phylogeny Group III 2009): Liliales, Asparagales, Poales, Ranunculales, Zygophyllales, Malpighiales, Fabales, Rosales, Geraniales, Malvales, Caryophyllales, Ericales, Gentianales, Solanales, Lamiales, Asterales, and Apiales.
**Life form**. Species were assigned to the following three major growth categories: woody plants, graminoid plants, and forbs.
**Adult longevity**. Species were grouped into two classes: annual (including a few biennials) or perennial.
**Seed mass**. The mean seed mass of each species was assigned to a class: < 0.1 mg, 0.1–0.3 mg, 0.31–1.0 mg, 1.1–3.0 mg, 3.1–9.0 mg, 9.1–30 mg, or > 30 mg (Moles et al., 2000).
**Seed shape**. Each species was classified into one of six categories based on our observations and relevant literature (Liu, 1992; Gordon, 1998; Liu et al., 2005, 2014): (1) spheroid or nearly spheroid; (2) cylindrical, tubular, or conical; (3) ellipsoid, broad-ellipsoid, narrow-ellipsoid, ovoid, elongate-ovoid, or obovoid; (4) trigonous or prismatic; (5) oblate-discoid, lenticular, or plane; or (6) fusiform, acicular, or linear.
**Dispersal model**. Species were classified into five groups according to the morphological features of their seeds: (1) zoochorous species, defined as having awns, spines, or hooks to adhere to animals (epizoochory), or having fleshly or arillate fruits for animals to eat (endochory); (2) anemochorous species, defined as having membranous wings, hairs, bracts, a persistent or inflated perianth, or a pappus; (3) ombrohydrochorous species, defined as those wherein the seeds produce mucilage upon being wetted; (4) autochorous species, which are ballistically dispersed from explosively dehiscing capsules that throw the seeds some distance from the parent plant; or (5) barochorous species, defined as those lacking any obvious dispersal mechanism or disperser reward (Navarro et al., 2009; Liu et al., 2014).
**Onset of flowering**. Species were assigned to one of three groups: (1) early, where flowering begins in May; (2) middle, where flowering begins in June; or (3) late, where flowering begins in July and August.
**Seed color**. Each species was classified into one of seven color categories based on our observations and relevant literature (Liu, 1992; Gordon, 1998; Liu et al., 2005, 2014): (1) light brown, brown, dark brown, or nut brown; (2) light reddish brown, reddish brown, or dark reddish brown; (3) light yellowish brown, yellowish brown, or dark yellowish brown; (4) pale yellow, yellow, orange, or reddish yellow; (5) light green, green, or dark green; (6) gray, grayish white, or grayish black; or (7) black.
**Ecotype**. Each species was assigned to an ecotype category based on the seed collection site: weed, steppe plant, meadow plant, or psammophyte.

## Results

### Patterns of seed mass and shape

**Figure 1:**
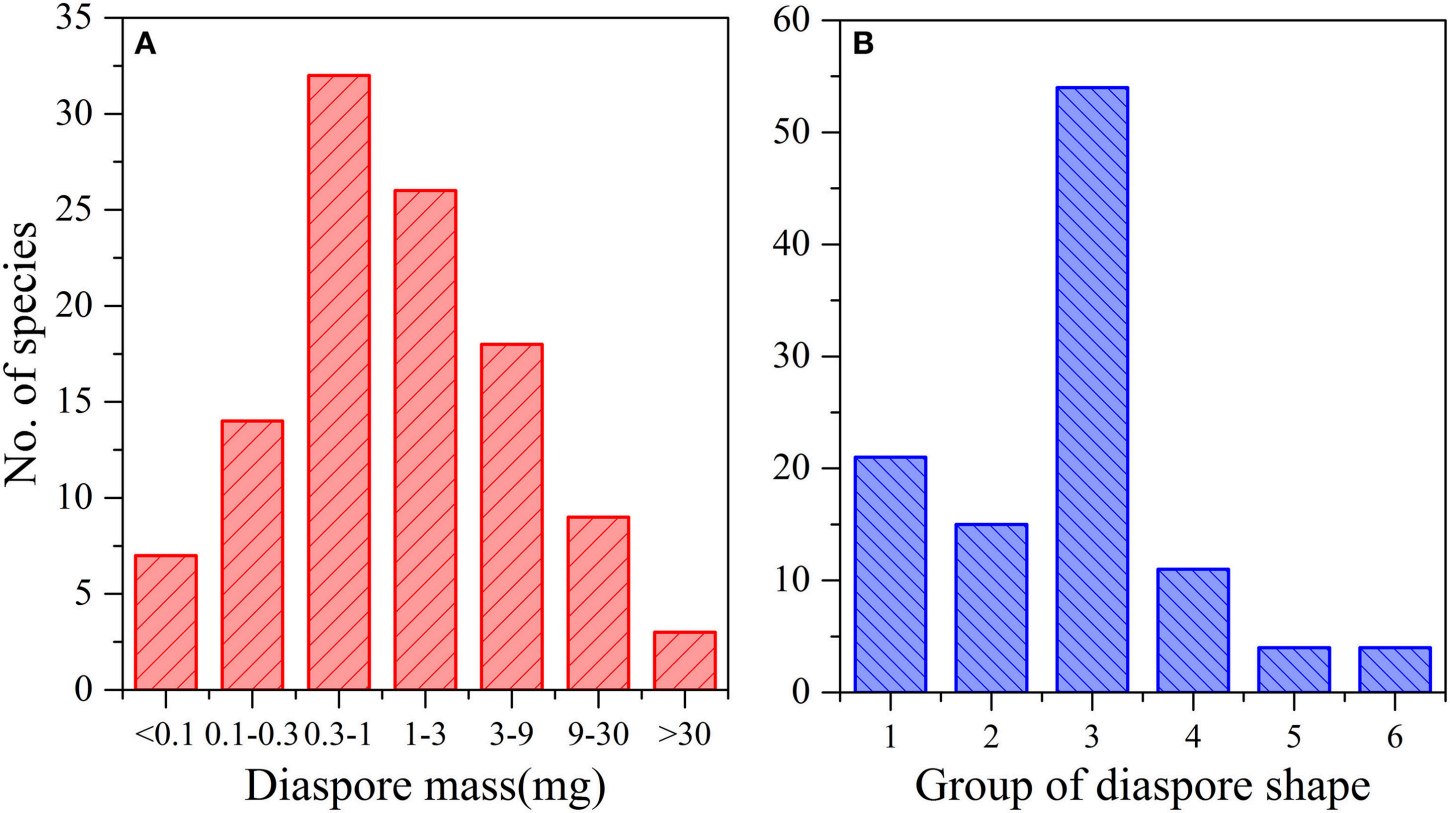
**Frequency distribution of seed mass (A) and seed shapes (B)**.

Seed mass ranged from 0.011 mg in *Solanum nigrum* L. to 130.758 mg in *Tribulus terrestris* L., with a mean of 4.988 mg. The frequency of seed mass classes had an approximately normal distribution (Figure 1A). Seven species had a weight of < 0.1 mg, 14 species of 0.1–0.3 mg, 32 species of 0.3–1.0 mg, 26 species of 1.1–3.0 mg, 18 species of 3.1–9.0 mg, 9 species of 9.1–30.0 mg, and 3 species of > 30 mg.

According to the calculated mean seed shape variance and direct observations, the seed shapes of 109 species could be divided into six groups, of which 68.8% (75 species) were close to spherical to ovoid (Figure 1B).

### Relationships between seed mass, seed shape, and GP

**Figure 2:**
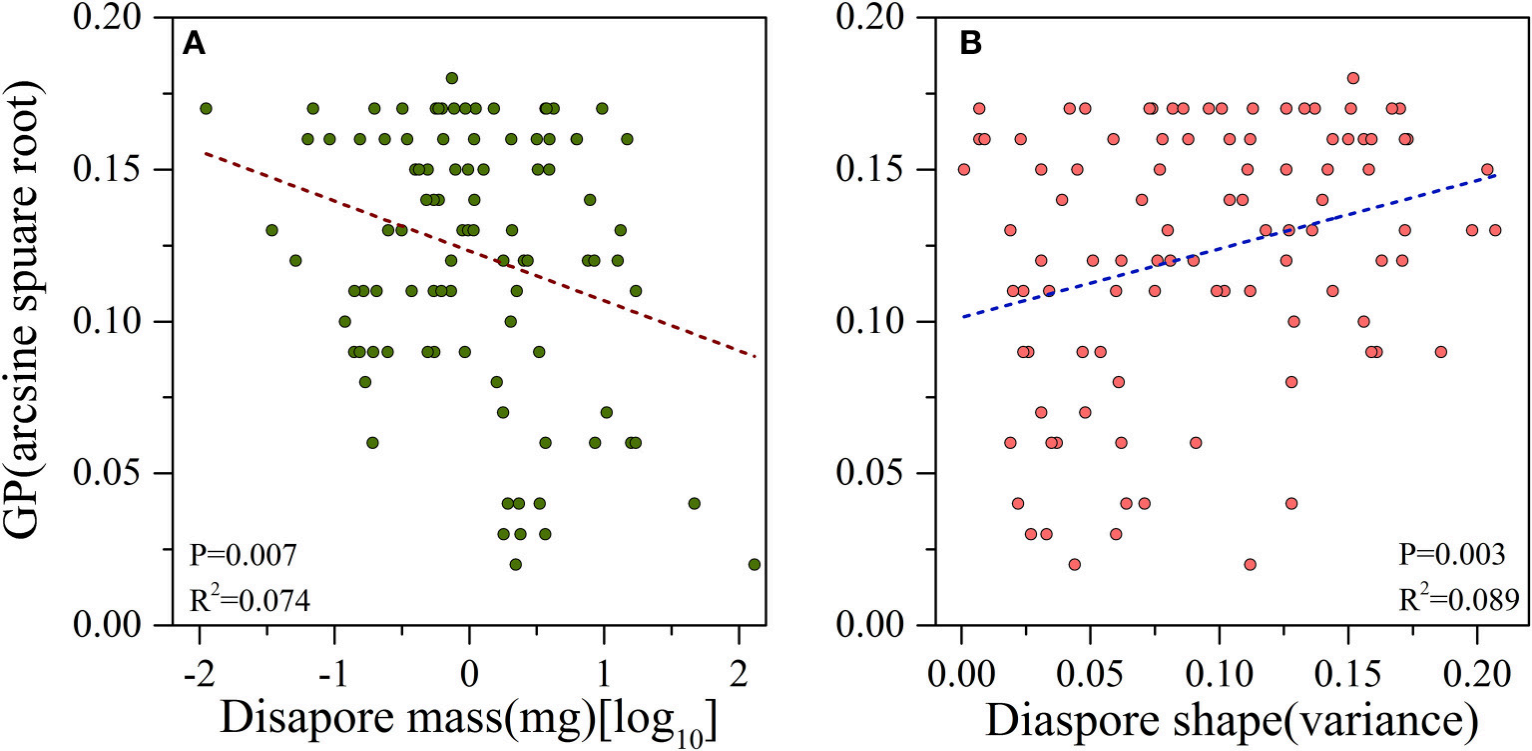
**Linear relationships between (A) log-transformed diaspore mass and arcsine square root-transformed germination percentage (GP); (B) variance of diaspore shape and arcsine square root transformed GP**.

**Figure 3:**
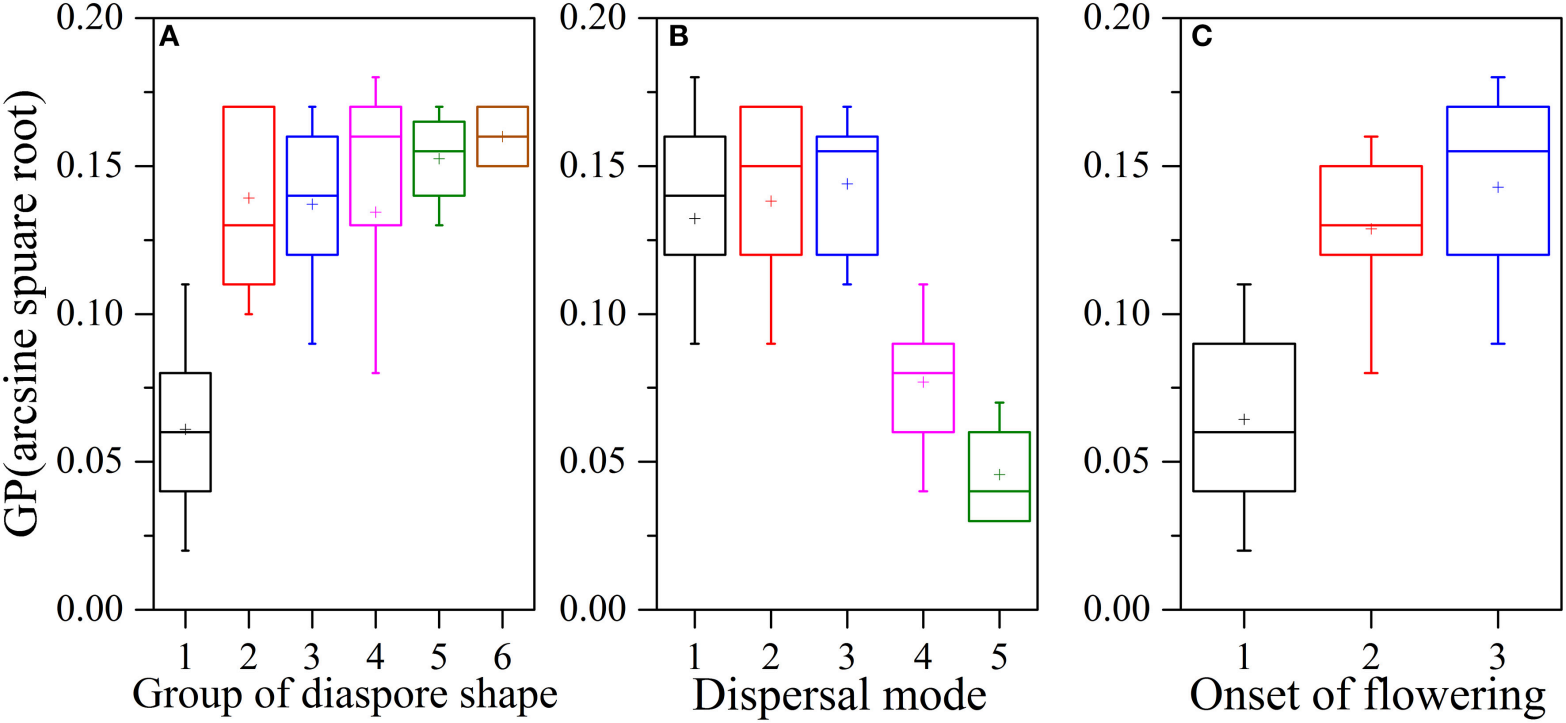
**Relationships between seed shape, dispersal type, onset of flowering and arcsine square root-transformed germination percentage**. Plots of **(A)** seed shape [(1) spheroid or nearly-spheroid; (2) cylindrical, tubular, or conical; (3) ellipsoid, broad-ellipsoid, narrow-ellipsoid, ovoid, elongate-ovoid, or obovoid; (4) trigonous or prismatic; (5) oblate-discoid, lenticular, or plane; (6) fusiform, acicular, or linear] and GP, **(B)** dispersal type [(1) zoochorous, (2) anemochorous, (3) ombrohydrochorous, (4) barochorous, (5) autochorous] and GP, and **(C)** onset of flowering [(1) early, where flowering begins in May; (2) middle, where flowering begins in June; (3) late, where flowering begins in July and August] and GP. Boxplots showing mean (+), median (—), quartiles, and outliers (−).

There was a significant negative correlation between seed mass and GP (*P* = 0.007, *R*^2^ = 0.074, Figure 2A), i.e., smaller seeds had higher germination percentages than larger ones. Seed shape was positively and significantly associated with germination percentage (*P* = 0.003, *R*^2^ = 0.089, Figure 2B), i.e., the elongate- and flat- seeded species had higher GPs than round-seeded species (Figure 3A).

### Effects of phylogeny and life history attributes on GP

A series of ANOVAs was used to determine whether the variance in GP among 95 species (excluding species with no germination and with only one representative per order) was correlated with the assessed factors. One-way ANOVAs showed that five factors (phylogenetic group, seed mass, seed shape, dispersal type, and onset of flowering) had statistically significant effects on GP (Table 1, Figures 3A–C). Dispersal type explained the highest percentage of the variation in GP when considered singly (61.2%), and onset of flowering, seed shape, phylogenetic group, and seed mass explained 60.6%, 59.9%, 35.5%, and 17.6% of the variation in GP, respectively (see Table 1). Further, the remaining four factors (life form, adult longevity, ecotype, and seed color) did not have significant effects and together explained 11.5% of the variation in GP (Table 1).

**Table 1:** **Results of one-way ANOVAs showing the effects of phylogenetic group, seed mass, seed shape, dispersal type, adult longevity, life form, onset of flowering, and ecotype on germination percentage among species**.

**Source of variation**	**df**	***F***	**Sig**.	***R*^2^**
Phylogenetic group	12	3.761	0.000^***^	0.355
Seed mass	6	3.126	0.008^**^	0.176
Seed shape	5	26.565	0.000^***^	0.599
Dispersal type	4	35.526	0.000^***^	0.612
Onset of flowering	2	70.779	0.000^***^	0.606
Adult longevity	1	0.564	0.454^ns^	0.006
Life form	2	0.054	0.947^ns^	0.001
Ecotype	3	1.733	0.166^ns^	0.054
Seed color	6	0.839	0.543^ns^	0.054

*df, degrees of freedom; R^2^, proportion of GP variance explained by each factor*.

** *P < 0.01*;

*** *P < 0.001*;

ns *P > 0.05*.

Two-way ANOVAs detected no significant interaction effects of any pair of factors that had separate effects on GP in the one-way ANOVAs (Table 2). In addition, the two-way ANOVAs revealed nonrandom associations between factors because the proportion of the total variance in GP explained by each of the factors (the *R*^2^ value) in the two-way ANOVAs was less than in the one-way ANOVAs (Table 2).

**Table 2:** **Results of two-way ANOVAs showing the independent effects of one of two main factors that have significant effects in one-way ANOVAs, and interaction effects of the pairs of factors on germination percentage**.

**Source of variation**	**Effect of A**				**Effect of B**				**Effect of A** × **B**			
**(A/B)**	**df**	***P***	***F***	***R*^2^**	**df**	***P***	***F***	***R*^2^**	**df**	***P***	***F***	***R*^2^**
PG/SM	12	0.008^*^	2.613	0.274	6	0.163^ns^	1.609	0.084	23	0.975^ns^	0.470	0.095
PG/SS	12	0.095^ns^	1.676	0.085	5	0.000^***^	17.057	0.360	17	0.752^ns^	0.738	0.053
PG/DT	12	0.451^ns^	1.009	0.056	4	0.000^***^	16.499	0.306	15	0.991^ns^	0.319	0.022
PG/OF	12	0.351^ns^	1.131	0.058	2	0.000^***^	34.036	0.290	15	0.832^ns^	0.639	0.041
SM/SS	6	0.067^ns^	2.084	0.049	5	0.000^***^	18.512	0.362	16	0.103^ns^	1.567	0.098
SM/DT	6	0.196^ns^	1.484	0.035	4	0.000^***^	20.988	0.329	14	0.129^ns^	1.514	0.083
SM/OF	6	0.062^ns^	2.104	0.049	2	0.000^***^	47.329	0.365	9	0.064^ns^	1.902	0.066
SS/DT	5	0.109^ns^	1.878	0.037	4	0.033^*^	2.765	0.044	5	0.299^ns^	1.239	0.025
SS/OF	5	0.040^**^	2.454	0.045	2	0.000^***^	8.864	0.065	5	0.331^ns^	1.170	0.022
DT/OF	4	0.001^***^	5.425	0.081	2	0.000^***^	8.480	0.063	5	0.981^ns^	0.145	0.003

*df, degrees of freedom*.

* *P < 0.05*,

** *P < 0.01*,

*** *P < 0.001*,

ns *P > 0.05. PG, phylogenetic group; SM, seed mass; SS, seed shape; DT, dispersal type; OF, onset of flowering*.

Factorial ANOVAs revealed an independent effect of each factor on GP (Table 3). The difference between the proportion of the ss (*R*^2^ value) of the complete and incomplete models can represent the proportion of the ss explained by the removed factor (Table 3), so we could systematically estimate the source of variance in GP. Variance was explained by phylogeny (5.9%), dispersal type (4.9%), seed shape (3.1%), onset of flowering (2.4%), and seed mass (2.2%) (Table 3).

**Table 3:** **Factorial ANOVAs for the independent effects of each main factor and their associations**.

**Source of variation**	**df**	***F***	***P***	***R*^2^**
**COMPLETE MODEL**				
Phylogenetic group	12	1.573	0.122^ns^	0.058
Seed mass	6	1.212	0.312^ns^	0.022
Seed shape	5	2.006	0.089^ns^	0.031
Dispersal type	4	3.946	0.006^**^	0.049
Onset of flowering	2	3.858	0.026^*^	0.024
Model	29	8.923	0.000^***^	0.799
**PHYLOGENETIC GROUP REMOVED**				
Seed mass	6	1.290	0.272^ns^	0.026
Seed shape	5	1.761	0.131^ns^	0.030
Dispersal type	4	2.877	0.028^*^	0.039
Onset of flowering	2	7.212	0.001^***^	0.049
Model	17	12.955	0.000^***^	0.741
**SEED MASS REMOVED**				
Phylogenetic group	12	1.640	0.100^ns^	0.062
Seed shape	5	1.744	0.136^ns^	0.027
Dispersal type	4	3.513	0.011^*^	0.044
Onset of flowering	2	5.210	0.008^**^	0.033
Model	23	10.742	0.000^***^	0.777
**SEED SHAPE REMOVED**				
Phylogenetic group	12	440.898	0.172^ns^	0.057
Seed mass	6	1.433	0.465^ns^	0.019
Dispersal type	4	0.951	0.001^***^	0.065
Onset of flowering	2	4.922	0.001^***^	0.050
Model	24	9.669	0.000^***^	0.768
**DISPERSAL TYPE REMOVED**				
Phylogenetic group	12	1.112	0.365^ns^	0.048
Seed mass	6	0.824	0.555^ns^	0.018
Seed shape	5	2.622	0.031^*^	0.047
Onset of flowering	2	4.487	0.015^*^	0.032
Model	25	8.301	0.000^***^	0.750
**ONSET OF FLOWERING REMOVED**				
Phylogenetic group	12	2.063	0.032^*^	0.083
Seed mass	6	1.560	0.173^ns^	0.031
Seed shape	5	3.436	0.008^**^	0.058
Dispersal type	4	4.279	0.004^**^	0.057
Model	27	8.567	0.000^***^	0.775

*For each main factor, R^2^ is the proportion of the sum of squares attributed to the main effect. To calculate the proportion of the variance explained by only one of the main factors, the R^2^ of the incomplete model with the factor removed was subtracted from the R^2^ of the complete model. df, degrees of freedom*.

* *P < 0.05,

** *P < 0.01*,

*** *P < 0.001*,

ns *P > 0.05*.

## Discussion

**Figure 4:**
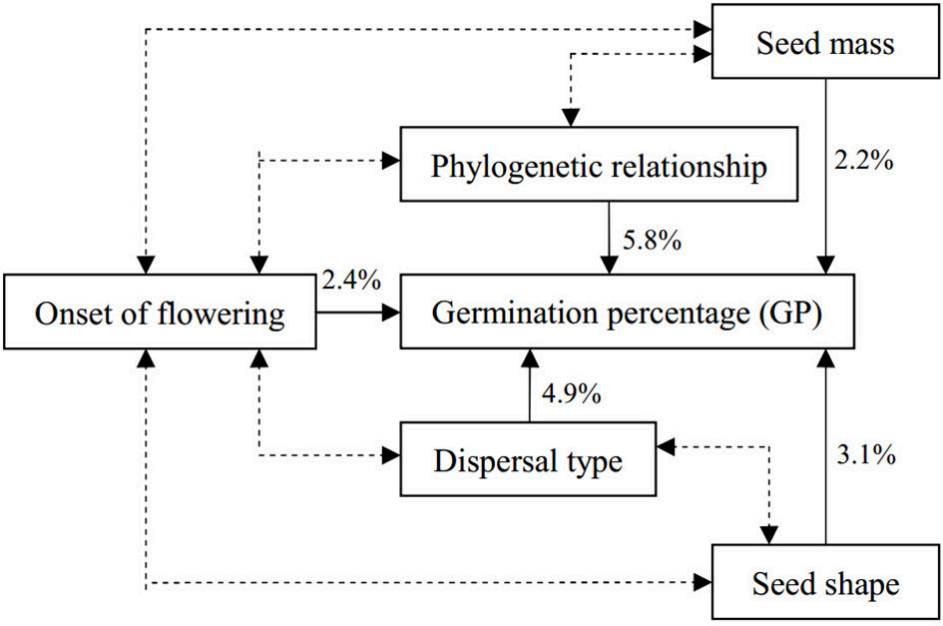
**Factorial model of factors explaining variation in GP among species in degraded sandy grassland, considering the smallest number of possible independent effects**. Percentages indicate the proportion of the variance explained independently by each factor. Double arrows indicate stronger associations between factors, see text.

GP in the degraded sandy grassland was affected by phylogeny, seed traits and ecological factors that interacted strongly in a factorial model. Figure 4 shows a model of the significant correlations found between mean GP and five independent variables analyzed in this study. Phylogenetic relatedness, dispersal type, and seed shape, in that order, were the three factors with the greatest contribution to the factorial model (14% of the variance in GP). Seed mass and onset of flowering combined explained only 4.6% of the variation in GP.

Several studies have advocated using phylogenetic correlates of plant species within a community to explain variance in ecological or other traits among taxa (Miles and Dunham, 1993; Figueroa and Armesto, 2001; Zhang et al., 2004; Norden et al., 2009; Xu et al., 2014). Our results clearly showed that phylogenetic groups significantly influenced GP for the species in the degraded sandy grassland. Previous research showed a phylogenetic pattern of seed germination in plant species of temperate rain forests (Figueroa, 2003), alpine meadows (Bu et al., 2007; Xu et al., 2014), and arid and semiarid zones (Wang et al., 2009). We think that variation in GP may be closely dependent on phylogeny, i.e., inherent traits of species may play a remarkable role in evolution. In Sheffield, England, germination rates of species in the same families were more similar than those of species in different families (Grime et al., 1981). Germination behavior might be more similar in closely related species than in distantly related species regardless of ecological factors. Our results suggest that phylogeny should be taken into account when investigating the role of natural selection in the regulation of germination or other life history attributes.

Our results indicated that GP was also correlated with seed traits such as shape and mass. The most significant and interesting finding of our study was the significantly negative relationship between GP and seed mass (Figure 1A, Table 1). This result was inconsistent with other studies that showed that seed mass was significantly positively correlated with GP (Vanmolken et al., 2005; Wu and Du, 2007; Galíndez et al., 2009). Traditionally, large and heavy seeds have better GP (Wu and Du, 2007), emergence, seedling survival, and growth than small seeds because of their larger reserves (Bonfil, 1998), which is consistent with the idea that heavy seed mass confers a competitive advantage, as assumed by the competition-colonization model (Rees et al., 2001). However, our results showed that small-seeded species have a higher GP than large-seeded ones in the degraded sandy grassland. There are several possible interpretations for the contradiction. Firstly, our study contained species with a range of growth forms from a wide range of families, and the species in Asteraceae, Poaceae, and Fabaceae were dominant in this study site. Asteraceae species had a lighter average seed mass (mean mass 1.59 mg; 73% were less than 1.00 mg in our study site) than the average of all species (mean mass 4.90 mg), and they had a higher GP (>80%). Legume seeds, which tend to have a hard seed coat, typically had a greater average seed mass (mean mass 11.30 mg) than species without a hard seed (4.30 mg). Hard seeds often require scarification to breach the test and do not immediately germinate (Smıkal et al., 2014). Secondly, the Horqin sandy land is characterized by Aeolian sandy soils (coarsely textured with a loose structure), and seeds seem to be more easily buried in sand ecosystems than in others. Small seeds may become more deeply buried in open, loose, sandy soil, reducing seed germination. Thirdly, certain small-seeded species in this study, such as *Chloris virgata* Sw.*, Euphorbia humifusa* Willd., *Datura stramonium* L., and *Solanum nigrum*, germinate immediately after dispersal ((Liu et al., 2004a), b). Finally, it is well known that environmental factors are crucial from seed germination (Tielbörger and Petrü, 2010; Cendán et al., 2013; Li et al., 2015). Germination strategy is can be considered a coevolutionary strategy to the extreme environmental conditions in the degraded sandy grasslands. Plants in the degraded sandy grassland of Horqin Sandy Land are exposed to a highly disturbed and stressed environment due to overgrazing, wind, and drought. The small-seeded species should have greater seed density, higher germination percentage, and lower seedling survival at potential recruitment sites (Liu et al., 2004a, 2007). The large-seeded species have greater seedling establishment but a lower seed density and germination percentage (Liu et al., 2004a,b; Moles and Westoby, 2004; Li et al., 2006; Liu et al., 2007), even under favorable conditions. Thus, small-seeded species tend to produce large number of light seeds for long-distance dispersal to escape from unfavorable spatial conditions, and large-seeded species tend to be dormant to spread the risk of encountering unfavorable temporal conditions. Similarly, Rees (1996) found that seed germination was negatively related to seed size in a British grassland community with high overgrazing and stated that this was consistent with heavy seeds having better establishment success and suffering higher levels of unfavorable conditions (Rees, 1996).

We found that species with elongate and flat seeds had higher GPs than species with round seeds (Figure 1B). Our results indicated that the pattern between seed shape and GP found by Grime et al. (1981) applies to our study region. Interestingly, our study indicated that GP is more strongly correlated with seed shape than with seed mass in the Horqin sandy land (Figure 1, Table 1). We hypothesize that phylogeny may be responsible for this pattern because seeds of Poaceae and Asteraceae, which are often elongated or flattened, tend to germinate easily (Grime et al., 1981; Liu et al., 2003).

Ecological factors, such as seed dispersal type and onset of flowering, significantly influenced GP and could independently explain 5.9% and 2.4% of the total variance in GP in our study area, respectively (Tables 1, 3). Seeds of well-dispersed species germinated more frequently (Figure 2A), with anemochorous, zoochorous, and ombrohydrochorous species having higher GPs than either barochorous or autochorous species (Figure 2A). These results are consistent with those reported by Bu et al. (2007). One possible interpretation for this relationship between GP and dispersal type is that seed dispersal may be adapted to avoid or minimize sibling competition and place seeds at “safe sites,” where they can successfully germinate and establish (Gutterman, 1994; Lu et al., 2013; Bolmgern and Eriksson, 2015). In most germinated seeds, restricted (barochorous) dispersal can lead to competition among the siblings produced by a fecund maternal plant (Cheplick, 1996; Fragoso et al., 2003). The dispersal potential of seeds with different dispersal types varies greatly, with both wind- and vertebrate-dispersed seeds potentially having a greater dispersal distance from the parent than seeds of barochorous and autochorous species. In some vertebrate-dispersed seeds, germination may be promoted by passage through the animal's digestive system (Navarro et al., 2009). Thus, seeds with long dispersal distances (anemochorous, zoochorous, and ombrohydrochorous species) may germinate more frequently than seeds with short-range unassisted dispersal (which are more likely to experience sibling competition). Early onset of flowering may be correlated with early dispersal and germination, and thus a longer period of juvenile growth. A long juvenile period that includes the unfavorable season increases the risk of mortality before reproduction. These selection pressures involve tradeoffs that may hinder simple, direct effects of onset of flowering on germination. Our results revealed that most of the late-flowering species belong to Poaceae and Asteraceae. These species had a higher GP in the degraded sandy grassland community, which explains why onset of flowering species had a significant effect.

In this study, we found that ecological factors such as ecotype, life form, and adult longevity had insignificant effects on GP. In other words, the difference in GP between woody, graminoid, and forb species, and between annuals and perennials, was not significant. Previous studies have presented conflicting evidence of the relationships between life form, adult longevity, ecotype, and seed germination strategies. For example, Xu et al. (2014) indicated that there were no significant effects of life form (between annual and perennial) on GP in eastern Tibetan plateau species (Xu et al., 2014), while Rees suggested that perennials tended to have higher germination percentage than annuals based on a large grass data set (Rees, 1994). Bu et al. (2008) found life form (woody, graminoid, and forb) and adult longevity (annual and perennial) had a significant effect on seed germination time in eastern Qinghai-Tibet plateau species (Bu et al., 2008). The effects of life form and adult longevity on seed germination might vary by habitat or flora.

In this study, we provided insights into correlations between seed germination and phylogeny, seed traits, and ecological factors in a degraded sandy grassland of Horqin Sandy Land. Our most important finding is that seed germination strategies in this habitat were affected by the interactions of phylogeny, seed traits, and ecological factors. Phylogeny could explain the biggest proportion in variance of GP, which suggests that there are strong phylogenetic constraints on germination. Smaller seeds, elongate and flat seeds, seeds with long dispersal distances (anemochorous, zoochorous, and ombrohydrochorous species), and seeds from late-flowering species had higher GPs. Moreover, we found that phylogeny had a close association and interaction with seed size, shape, and dispersal strategy (Tables 1–3). Thus, we suggest that phylogeny may constrain seed germination, and because seed mass, shape, dispersal, and flowering time may be related to phylogeny, they also constrain germination. Therefore, phylogeny and life-history attributes could not be considered separately. Seed germination, like any other trait, is shaped by short-term selective pressures and long-term phylogenetic constraints.

## Conclusion

In this study of the Horqin Sandy Land, a degraded sandy grassland in northeastern Inner Mongolia, China, we found that (1) GP was negatively and significantly correlated with seed mass, (2) species with elongate and flat seeds had higher GPs than species with round seeds, and (3) phylogenetic relatedness, dispersal type, and seed shape, in that order, were the three factors with the greatest contribution to the complete model (14% of the variance in GP). Furthermore, (1) GP, like any other trait, is shaped both by short-term selective pressures and long-term phylogenetic constraints, and (2) comprehensive studies at the community level that use multivariate approaches are needed to enhance our understanding of the evolutionary and ecological forces shaping germination strategies.

## Author contributions

BL designed the research and collected and analyzed the data. ZW analyzed the data and drafted the manuscript. LW and ZL revised English language and contributed to paper writing. QL and YL assisted with data collection. All authors took part in writing the manuscript.

## Funding

Funding was provided by the Key Project of Chinese National Programs for Fundamental Research and Development (No. 2013CB429903) and National Natural Science Foundation of China (No. 31570448) and the New Century Excellent Talents Program of Fujian Province (K8015053A).

### Conflict of interest statement

The authors declare that the research was conducted in the absence of any commercial or financial relationships that could be construed as a potential conflict of interest.
